# Application of attenuation coefficient in the assessment of hepatic involvement in children and adolescents with Wilson’s disease

**DOI:** 10.1186/s12880-023-00979-7

**Published:** 2023-02-04

**Authors:** Jiajia Wang, Jinping Wang, Han Wang, Boqi Li, Yixing Wang, Lanting Sun, Xiaoqian Wu

**Affiliations:** 1 Department of Ultrasound, The First Affiliated Hospital of Anhui University of Chinese Medicine, 117 Meishan Road, Shushan District, Hefei, 230031 Anhui China; 2 Department of Encephalopathy, The First Affiliated Hospital of Anhui University of Chinese Medicine, Hefei, China

**Keywords:** Wilson’s disease, Attenuation coefficient, Shear wave measurement, Liver stiffness, Hepatocyte steatosis

## Abstract

**Background:**

To investigate whether the attenuation coefficient (ATT) can be used as a noninvasive index to assess liver involvement in children and adolescents with Wilson’s disease (WD).

**Methods:**

Children and adolescents diagnosed with WD were retrospectively collected from the First Affiliated Hospital of the Anhui University of Traditional Chinese Medicine between May 2022 and August 2022. The findings on ATT, Shear Wave Measurement (SWM), AST to platelet ratio index (APRI), and fibrosis 4 (FIB-4) score were obtained. The liver involvement of WD was classified into 3 groups based on serum levels of collagen type IV (CIV), hyaluronic acid (HA), laminin (LN) and precollagen type III N-terminal peptide (PIIINP): (1) Group1 (n = 25), no abnormalities in CIV, HA, LN and PIIINP; (2) Group2 (n = 19), elevation of 1 or 2 indexes in CIV, HA, LN, and PIIINP; Group3 (n = 18), elevation of 3 or 4 indicators in CIV, HA, LN, and PIIINP. The levels of ATT, SWM, APRI and FIB-4 were compared between the 3 groups; and correlation of ATT with SWM and triglyceride (TG) was performed using Spearman's correlation analysis. The Receiver operating characteristic (ROC) curve was used to analyze the diagnostic efficacy of ATT alone and its combination with SWM, APRI, and FIB-4 in children and adolescents with WD.

**Results:**

A total of 62 children and adolescents with WD were retrospectively retrieved. ATT levels were significantly different in intergroup comparisons (*P* < 0.001). The ROC curve showed that the area under the curve (AUC) for the diagnosis of hepatic steatosis using ATT was 0.714, 0.712 and 0.867 in Group 1 versus Group 2, Group 2 versus Group 3, and Group 1 versus Group 3, respectively; the sensitivity for the diagnosis of hepatic steatosis in Group 1 versus Group 2 was 89.47% with the cutoff value of ATT of 0.73 dB/cm/MHz. No significant correlation found between ATT and TG (*ρ* = 0.154, *P* = 0.231). Compared to ATT alone, the combination of ATT with APRI and FIB-4 or the combination of ATT with SWM, APRI, and FIB-4 showed a better diagnostic efficacy in Group 1 versus Group 2 (both *P* = 0.038).

**Conclusion:**

ATT could be used as a non-invasive index for the evaluation of liver steatosis in children and adolescents with WD, with a good clinical applicative value. Furthermore, ATT in combination with APRI, FIB-4, and SWM might have better diagnostic efficacy than ATT alone.

## Background

Wilson's disease (WD), also known as hepatolenticular degeneration, is an autosomal recessive genetic disorder due to a mutation of the ATP7B gene that results in impaired liver copper excretion and accumulation of copper in tissues [1]. The liver is the main organ for copper metabolism [2], and the pathogenesis of WD is a direct consequence of the accumulation of copper in hepatocytes, initiated with hepatocellular steatosis and followed by chronic inflammation, liver fibrosis, and liver cirrhosis [3]. Liver biopsy is recognized as the gold standard for the assessment of pathological changes in the liver. However, most patients with WD present with a spectrum of neurologic manifestations, such as tremor, bradykinesia, rigidity, dystonia, chorea, dysarthria, and dysphagia [4], making invasive liver biopsy difficult to implement. Therefore, it is urgent to explore noninvasive techniques for the assessment of pathological changes in patients with WD.

Previous studies have shown that various ultrasonographic techniques, including two-dimensional(2D) ultrasound [5], transient elastography (TE), and two-dimensional shear wave elastography (2D-SWE), can be used to assess the liver fibrosis and liver cirrhosis process in patients with WD [5–7]. For patients with WD-associated liver cirrhosis, 2D ultrasound is able to display an unsmooth liver envelope, as well as abnormalities in the internal echogenicity of the liver. When the disease progresses to liver fibrosis or even liver cirrhosis, TE and 2D-SWE often suggest a subsequent increase in liver stiffness measurements. As reported, in pediatric patients with WD, the optimal threshold of liver stiffness for liver fibrosis is 8.30 Kpa by TE [8] and 8.50 Kpa by 2D-SWE [7]. However, previous studies have focused mainly on the stages of liver fibrosis and liver cirrhosis in patients with WD, and have rarely paid attention to noninvasive evaluations of hepatocellular steatosis, which is the most common early pathological change for WD in children and adolescents [9, 10].

In this study, we were aimed to find out whether there is a noninvasive examination that can evaluate the degree of fatty infiltration in liver tissues of WD patients, especially in children and adolescents, so as to further quantitatively analyze the degree of hepatocyte steatosis in these patients.

Recently, a new ultrasound method was developed in which attenuation coefficient (ATT) measurements are obtained from the ultrasound-based modalities for the quantitative measurement of fat in the liver, and it has been gradually applied in clinical practice [11]. Previous studies have demonstrated that ATT can be used to quantitatively assess liver fat content in patients with nonalcoholic fatty liver disease (NAFLD) and chronic liver disease, and is closely related to the time of onset and progression of liver lesions [12]. In clinical practice, ATT is preferred by children and adolescents with WD due to their noninvasiveness, high reproducibility, and high localization accuracy by 2D ultrasound.

Thus, in this retrospective study, our objective was to investigate the diagnostic value of the noninvasive index ATT in assessing liver involvement and quantifying the degree of liver steatosis in children and adolescents with WD.

## Methods

### Patients

This is a retrospective observational study. Children and adolescents diagnosed with WD were retrospectively collected from The First Affiliated Hospital of Anhui University of Chinese Medicine between May 2022 and August 2022. The inclusion criteria were the following: (1) patients diagnosed with WD according to the Leipzig diagnostic criteria proposed by the European Association for the Study of the Liver (EASL) [13]; (2) patients aged ≤ 18 years. The exclusion criteria were as follows: (1) patients with severe neurological impairments and difficulty controlling their breathing or movement of the limb during ultrasound, resulting in the failure of the ATT and/or SWM; (2) patients with absent or partially absent results of serologic indicators during the diagnosis and treatment of WD.

According to the inclusion and exclusion criteria, a total of 62 children and adolescents with WD were included. We inquired and recorded the duration of WD from diagnosis to time. Meanwhile, we recorded the classification of the WD patients according to organ involvement.

All procedures performed in this study were approved by the ethics committee of the Anhui University of Traditional Chinese Medicine (No. 2018AH-08).

### The calculation of laboratory indexes

The levels of indicators for blood routine, including platelet counts (PLT), alanine transaminase (ALT), aspartate transaminase (AST), triglyceride (TG), and ceruloplasmin (CP) were collected. The serological indexes for the non-invasive assessment of liver fibrosis, including the fibrosis 4 (FIB-4) score [14] and the AST to platelet ratio index (APRI) [15], were also calculated. The APRI values were calculated using the equation (AST/upper limit of normal) × 100/platelet count (10^9^/L). The upper limit of normal for AST was 40 U/L. The FIB-4 values were calculated using the equation age (years) × AST (U/L)/(platelets [10^9^/L] × [ALT (U/L)]^1/2^). Meanwhile, the 24-urinary copper excretion of WD patients were detected and recorded before and after the therapy.

### The examination of conventional ultrasound

The conventional ultrasound examination was performed using the Arietta 850 ultrasound instructions (Hitachi Medical, Tokyo, Japan) with a C715 convex array probe (frequency: 1–5 MHz). On the day of the abdominal ultrasound examination, the patient was fastened for at least 8 h before detection of the size of the liver and the internal echoes. The internal diameter of the portal vein and the velocity of the blood flow of the portal vein were measured in a standard section while the patient breathed calmly.

### The measurements of ATT and SWM

The measurements of ATT and SWM were performed immediately after the conventional abdominal ultrasound examination using the same machine. The ATT and SWM measurements were performed by experienced radiologists (5-year experience of abdominal ultrasound) who had performed at least 50 liver ATT and SWM measurements in the last six months. The patient was in a supine position (or in a left-lateral position if the image quality was poor), and the operator placed the probe perpendicular to the liver capsule in the right intercostal space, avoiding large intrahepatic vessels, and selected the right lobe of the liver (S5 segment was preferred, followed by S7 or S8) for the measurements, with the sampling frame positioned 1–2 cm below the liver capsule. The patient was instructed to hold his breath for 3–5 s following calm breathing. During the measurement, the operator's hand should remain still to make the image stable for more than 3 s. Subsequently, the update key was activated, and the machine was automatically measured, analyzed, and stored the images in 2 s, and a single measurement of ATT (dB/cm/MHz) and SWM (m/s) was automatically recorded. The ATT and SWM measurements would be repeated 5 times and the median would be taken for each patient.

### Quality control

In the ATT and SWM measurements, 2 indexes were used for quality control. (1) VsN: As the quality control indicator for a single measurement, VsN ≥ 50% suggests a valid result; (2) the interquartile range (IQR)/median: The measurement would be repeated 5 times for each patient, and the ratio of IQR to the median ≤ 30% suggests a valid result.

### Clinical stratification of liver involvement

The venous blood of the patients was recovered for the measurement of the liver fibrosis-related indexes, including collagen type IV (CIV, normal range: ≤ 95 ng/ml), hyaluronic acid (HA, normal range: < 120 ng/ml), laminin (LN, normal range: < 130 ng/ml), and N-terminal peptide type III of precollagen (PIIINP, normal range: < 15 ng/ml). WD liver involvement was classified into 3 groups based on laboratory findings: Group 1, no abnormalities in CIV, HA, LN and PIIINP; Group 2, elevation of 1 or 2 indexes in CIV, HA, LN and PIIINP; Group 3, elevation of 3 or 4 indicators in CIV, HA, LN and PIIINP.

### Therapy and response to the therapy

All WD patients received de-coppering therapy. Patients received sodium dimercaptosulphonate (DMPS) intravenous drip at a dose of 10–20 mg/kg/d, once a day. Consecutive 6 days was a course of treatment, the interval of 2 days can be repeated for multiple courses of treatment during hospitalization. The response to therapy was evaluated according to the of 24-urinary copper excretion.

### Statistical analysis

Statistical analysis was performed using SPSS (version 25.0) and MedCalc (version 12.7.0). Data with normal distribution were expressed as mean ± standard deviation (SD); Differences within groups were compared using analysis of variance (ANOVA), and the differences between groups were compared using independent test. Data with a skewed distribution were expressed as median (interquatile range [IQR]); Differences between groups were compared using *Kruskal–Wallis test*, and differences between groups were compared using the *Mann–Whitney U test* with Bonferroni correction. Categorical data were expressed as numbers (percentage), and the *chi-square test* was used for comparison between groups. Box plots were plotted for the ATT values of the 3 groups of patients with WD. Spearman correlation analysis was performed to assess the correlation of ATT with SWM and TG. The area under the operating characteristic curve (AUROC), the cut-off value (determined by the Yoden index), the sensitivity, and the specificity were calculated to reflect the diagnostic performance of ATT. The ROC curve was used to estimate the diagnostic efficacy of ATT combined with SWM, APRI, and FIB-4. Differences in diagnostic efficacy between ATT alone and combined indexes were compared using the DeLong test. *P* < 0.05 was considered statistically significant.

## Results

### Baseline characteristics of patients with WD

A total of 62 patients were included in this study, with a median age of 14.0 years (min–max: 5–18 years), the duration of WD from diagnosis ranged from 4 months to 16 years. There were 54 WD patients with hepatic involvement (85.5%, 53/62), 8 WD patients with neurological involvement (11.3%, 7/62) and 3 WD patients with both hepatic and neurological involvement (3.2%, 2/62). The baseline characteristics of the WD patients were shown in Table 1. Depending on the categories of liver involvement, the patients were divided into 3 groups according to the levels of CIV, HA, LN and PIIINP: Group 1 (n = 25), Group 2 (n = 19), and Group 2 (n = 18).

**Table 1:** Baseline characteristics of patients with WD

Parameter	All	Group 1	Group 2	Group 3	P*
Number (N)	62	25	19	18	–
Age (Year)	14.00 (5.00)	15.00 (4.50)	12.00 (6.00)	12.50 (5.25)	0.035
Male (N)	37	17	12	8	0.279
CP (g/l)	0.019 (0.022)	0.018 (0.026)	0.017 (0.018)	0.024 (0.024)	0.915
24 h urinary Cu-before (µg/24 h)	738.63 (513.47)	758.67 (480.58)	708.98 (460.43)	714.88 (715.17)	0.917
24 h urinary Cu-after (µg/24 h)	807.53 (503.56)	839.79 (426.14)	788.26 (1053.55)	777.24 (599.79)	0.198
CIV (ng/ml)	62.98 (31.29)	49.31 (32.18)	68.07 (21.01)	78.10 (68.90)	0.002
HA (ng/ml)	87.63 (63.30)	63.04 (24.04)	90.34 (41.77)	156.04 (91.67)	< 0.001
LN (ng/ml)	132.02 (95.84)	89.68 (49.80)	165.26 (59.20)	213.10 (92.36)	< 0.001
PIIINP (ng/ml)	34.40 (23.01)	30.82 (20.16)	30.01 (17.13)	45.91 (35.69)	0.008
PLT (×10^9^)	243.46 (119.00)	229.00 (81.00)	284.00 (111.00)	218.50 (155.00)	0.360
ALT (U/L)	49.90 (35.15)	49.30 (26.05)	68.60 (59.60)	36.40 (28.38)	0.008
AST (U/L)	34.35 (19.17)	28.00 (20.15)	37.60 (15.40)	33.35 (19.85)	0.036
TG (mmol/L)	1.32 (1.06)	1.26 (0.76)	1.46 (1.35)	1.51 (1.31)	0.648
Internal diameter of the portal vein (mm)	10.00 (2.10)	10.00 (3.00)	9.70 (2.50)	9.75 (1.50)	0.383
Velocity of the blood flow of the portal vein (cm/s)	19.90 (6.10)	20.70 (5.15)	20.10 (6.40)	18.50 (5.40)	0.383

Quantitative data were expressed with median (IQR)

*Kruskal–Wallis test or chi-squre test

### Comparison of serological and ultrasound indexes

**Fig. 1:**
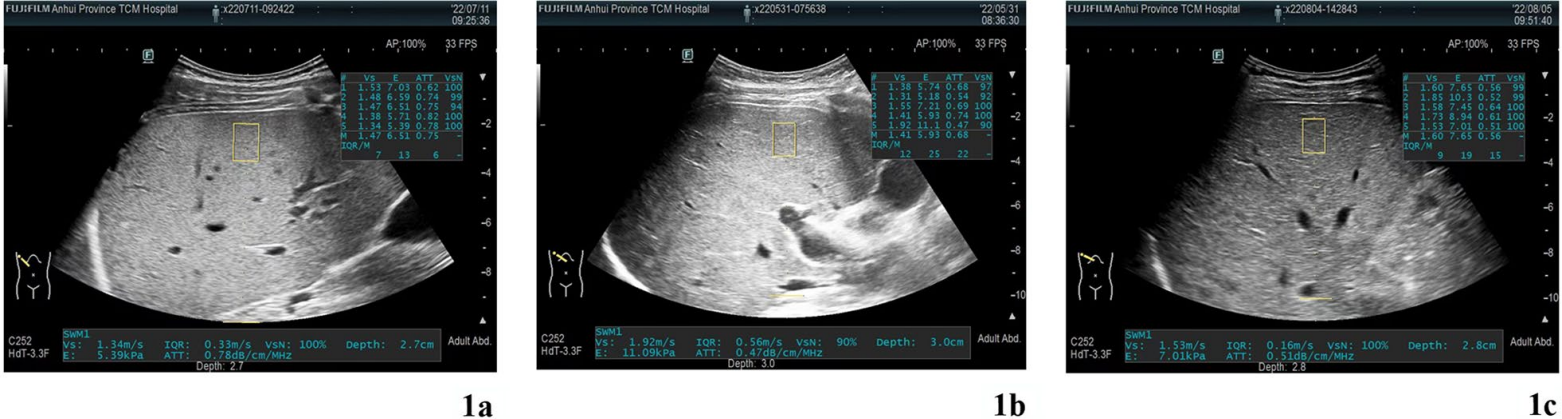
ATT ultrasound images for 3 groups of patients with WD (**a** Group1, median of ATT is 0.75; **b** Group1, median of ATT is 0.68, **c** Group1, median of ATT is 0.56)

**Fig. 2:**
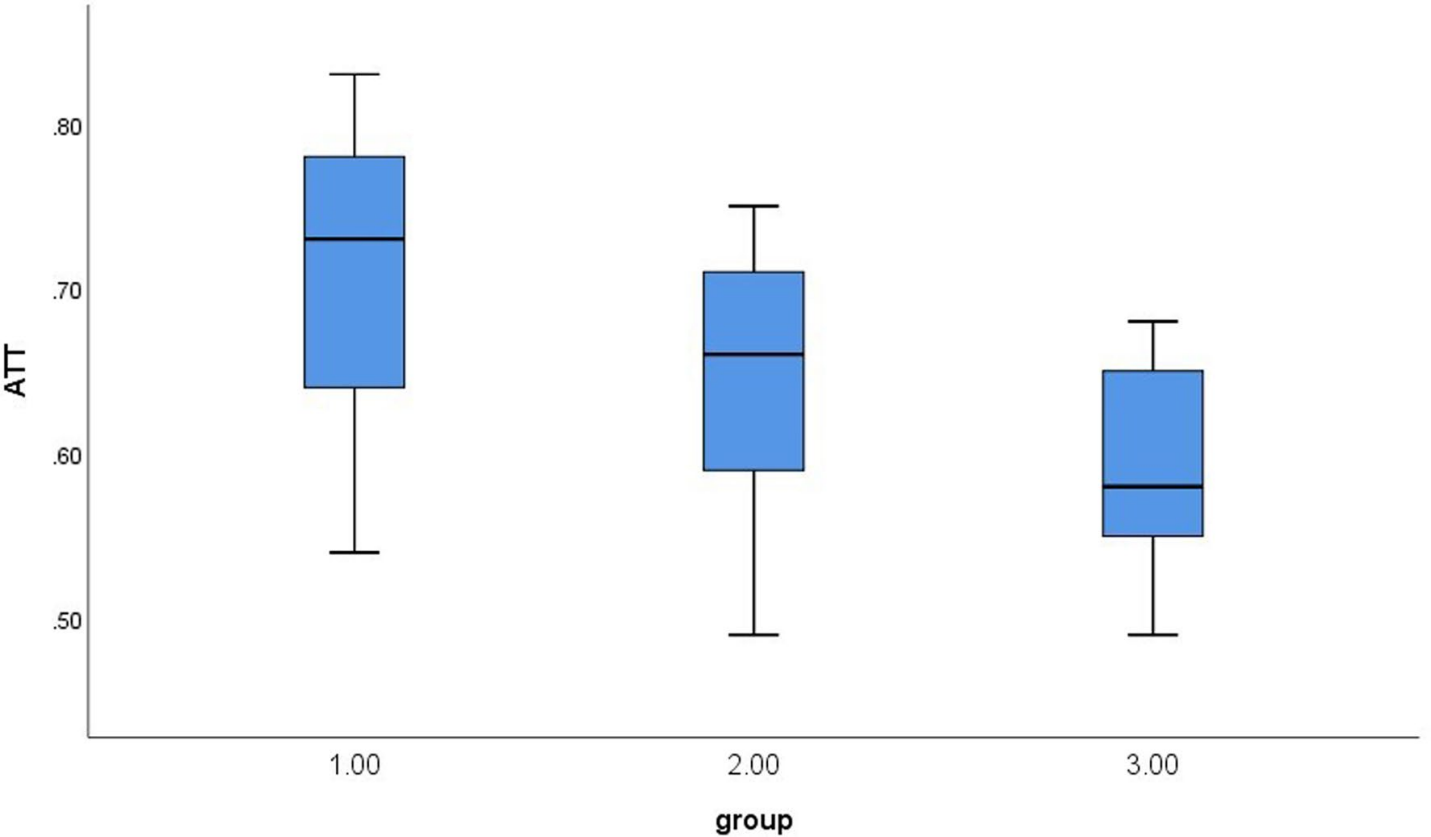
Box plots of ATT for 3 groups of patients with WD

The level of ATT was significantly different within three groups (*P* < 0.001). Compared to each other, the levels of FIB-4, APRI, and SWM were not significant different within three groups (*P* > 0.05) (Table 2). ATT measure images for each group were shown in Fig. 1 and ATT box plots were drawn for each group (Fig. 2). Significant differences were found in intergroup comparisons of ATT levels (Table 3).

**Table 2:** Results of serological indexes and ultrasound indexes in patients with WD

Parameter	All	Group 1	Group 2	Group 3	P*
Number (N)	62	25	19	18	–
APRI	0.37 (0.32)	0.33 (0.36)	0.42 (0.25)	0.37 (0.48)	0.608
FIB-4	0.28 (0.26)	0.31 (0.25)	0.25 (0.14)	0.28 (0.43)	0.476
SWM (m/s)	1.48 (0.55)	1.47 (0.45)	1.50 (0.56)	1.57 (0.82)	0.279
ATT (dB/cm/MHz)	0.66 (0.14)	0.73 (0.15)	0.66 (0.13)	0.58 (0.10)	< 0.001

Quantitative data were expressed with Median (IQR)

*Kruskal–Wallis test or chi-square test

**Table 3:** Inter-group comparison of ATT levels in patients with WD

ATT	*Z*	P*
Group 1 versus Group 2	− 2.408	0.016
Group 1 versus Group 3	− 4.069	0.000
Group 2 versus Group 3	− 2.206	0.026

*Mann–Whitney U test with Bonferroni correction

### Correlation of ATT with SWM and TG

**Fig. 3:**
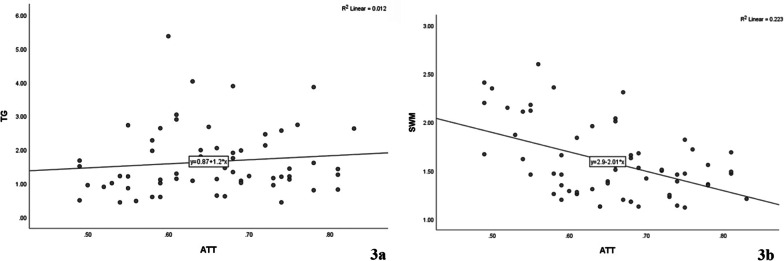
Correlation analysis of ATT with SWM and TG (**a** Spearman correlation of ATT and TG; **b** Spearman correlation of ATT and SWM)

There was no significant correlation between the levels of ATT and TG (*ρ* = 0.154, *P* = 0.231, Fig. 3a) and a weak negative correlation was found between ATT and SWM (*ρ* =  − 0.374, *P* = 0.003, Fig. 3b).

### Assessment of diagnostic efficacy of ATT

**Fig. 4:**
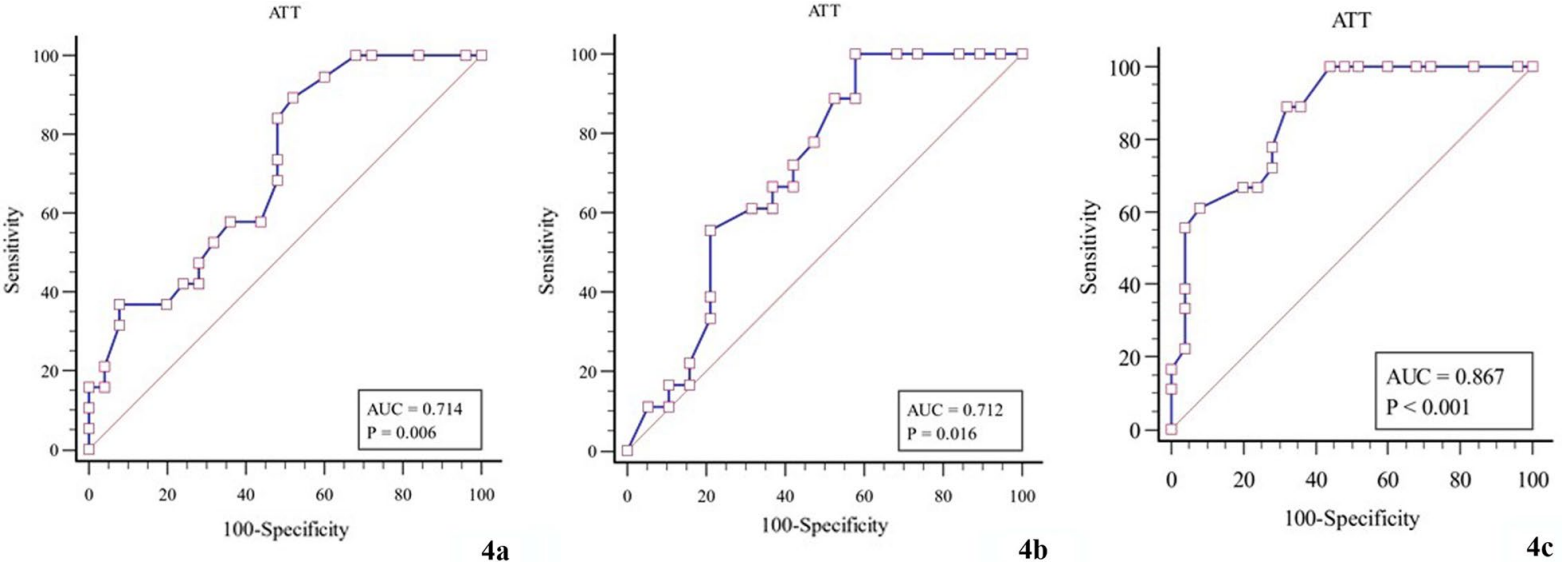
Diagnostic efficacy of ATT (**a** Group 1 vs. Group 2; **b** Group 2 vs. Group 3; **c** Group 1 vs. Group 3)

ROC curves were plotted to assess the diagnostic efficacy of ATT in the three groups of patients with WD. AUROC cut-off values, sensitivity, and specificity were calculated, respectively (Table 4, Fig. 4).

**Table 4:** Assessment of the diagnostic efficacy of ATT

Group	AUC	Cut-off value	Sensitivity (%)	Specificity (%)	*P*
Group 1 versus Group 2	0.714	0.73	89.47	48.00	0.006
Group 2 versus Group 3	0.712	0.68	100.00	42.11	0.016
Group 1 versus Group 3	0.867	0.66	88.89	68.00	< 0.001

### Assessment of diagnostic efficacy of ATT combined with SWM, APRI, and FIB-4

**Fig.5:**
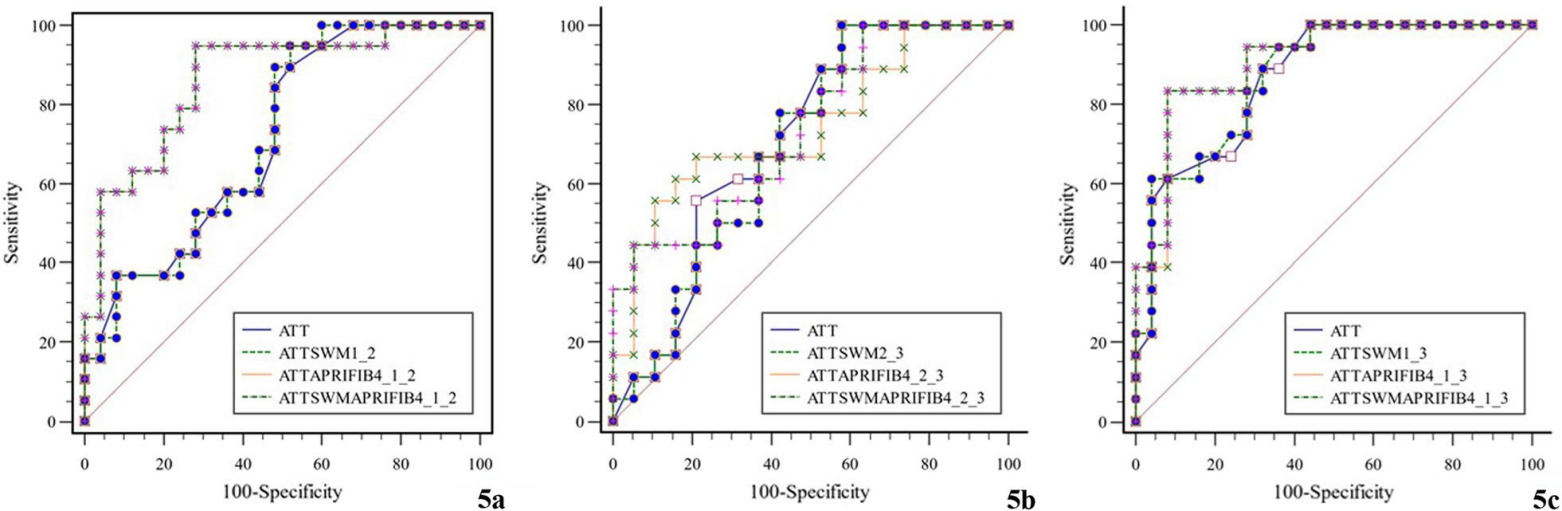
Assessment of diagnostic efficacy of ATT combined with other indexes (**a** Group 1 vs. Group 2; **b** Group 2 vs. Group 3; **c** Group 1 vs. Group 3)

The diagnostic efficacy of ATT combined with APRI and FIB-4 and that of ATT combined with SWM, APRI and FIB-4 were increased compared to ATT alone in Group 1 versus Group 2 (both *P* = 0.038). The diagnostic efficacy of ATT combined with other indexes was not statistically different compared to ATT alone (Table 5, Fig. 5).

**Table 5:** Assessment of diagnostic efficacy of the indexes alone

Group	Parameter	AUC	95%CI	P*
Group 1 versus Group 2	ATT + SWM	0.718	0.562–0.843	0.480
	ATT + APRI + FIB-4	0.863	0.726–0.948	0.038
	ATT + SWM + APRI + FIB-4	0.863	0.726–0.948	0.038
Group 2 versus Group 3	ATT + SWM	0.696	0.523–0.836	0.393
	ATT + APRI + FIB-4	0.743	0.573–0.872	0.643
	ATT + SWM + APRI + FIB-4	0.737	0.566–0.867	0.724
Group 1 versus Group 3	ATT + SWM	0.871	0.734–0.954	0.507
	ATT + APRI + FIB-4	0.909	0.781–0.975	0.231
	ATT + SWM + APRI + FIB-4	0.911	0.784–0.976	0.215

* the DeLong test

## Discussion

In this study, we analyzed the value of the ATT technique in the evaluation of liver involvement in children and adolescents with WD. To our knowledge, this is the first time the ATT technique has been applied to patients with WD, and the results showed that ATT had a better diagnostic performance compared to APRI, FIB-4, and SWM in the assessment of early liver involvement in children and adolescents with WD, and its diagnostic efficacy was improved when combined with other indices.

The level of SWM could be obtained simultaneously in ATT measurements in WD patients, and the results showed that the differences in SWM were not statistically significant in the three groups of WD patients (*P* = 0.270). Hwang et al. [16] also grouped the degree of liver involvement in WD patients based on clinical criteria. However, they concluded that 2D-SWE could have a more comparable clinical value than APRI and FIB-4. The inconsistent results may be attributed to the different age of the patients with WD included in the studies. WD patients in Jisum Hwang's study were between 3 and 35 years old, suggesting that a certain number of adult patients were included; while patients in our study were between 5 and 18 years old, without adult patients included. Furthermore, it has been noted that measurements of liver stiffness would be affected if included patients have moderate to severe hepatocellular steatosis, most of this change were in children of WD, which may also contribute to the poor performance of SWM in the present study [17]. Since Jisum Hwang et al. did not adopt the ATT technique, the comparison of the diagnostic value between ATT and 2D-SWE in patients with WD patients were unavailable in his study. In our study, the ROC curve showed that the AUC for the diagnosis of hepatic steatosis using ATT was 0.714, 0.712 and 0.867 in Group 1 versus Group 2, Group 2 versus Group 3, and Group 1 versus Group 3, respectively; the sensitivity for the diagnosis of hepatic steatosis in Group 1 versus Group 2 reached 89.47% with the cut-off value of ATT of 0.73 dB/cm/MHz. These results were consistent with those of a previous study, which calculated the median (95% confidence interval) of ATT values for each grade of steatosis (determined by histological examination of the liver biopsy and scored according to the NAFLD activity score: S0, < 5%; S1, 5–33%; S2, 33–66%; and S3, > 66%), and reported that the value of S2 was 0.72 (0.56–0.76) and the sensitivity for the diagnosis of S ≥ 2 to be 77.8% when ATT cutoff was 0.72 dB/cm/MHz [18]. Additionally, Nobuharu Tamaki et al. also used ATT to classify the degree of liver steatosis in 285 patients with HCB and NAFLD, and confirmed the value of ATT in the evaluation of liver steatosis [19]. However, given the rarity of disease and the infeasibility of liver biopsy, previous studies have never enrolled patients with WD in previous study. In this study, considering that WD is a hereditary disease with early hepatocellular steatosis during children and adolescence, we included WD patients under 18 years of age, analyzed their pathological changes in the liver and demonstrated that ATT is a better non-invasive tool for the evaluation of liver involvement. There is a limitation that WD Patients were increasingly diagnosed in children who are less than 3 years-old [10], the minimum age of the subjects in this study was 5 years old, and younger children were not included. Maybe it’s a reason that age existed the significant difference among the three groups in our study. Different age range whether have different degree of liver injured is determined by numerous factors, which may need the research with larger sample size of subjects in the future. In clinical practice, the degree of liver steatosis in patients with WD is often assessed using multiple indexes. Therefore, we combined ATT with three non-invasive indexes commonly used for the evaluation of liver fibrosis (SWM, APRI and FIB-4), and proved that the diagnostic efficacy for the early stages of liver involvement was improved compared to ATT alone (S1 vs. S2), which is consistent with the conclusion proposed from a previous study [20] where the area under the curve (AUROC) for the diagnosis of liver fibrosis using TE, 2D-SWE, ARPI and FIB-4 were 0.955, 0.842 and 0.897, 0.856, respectively; however, AUROCs were all increased in different combinations of indexes (0.961 for TE + ARPI, 0.911 for 2D-SWE + ARPI, 0.969 for TE + FIB-4, and 0.931 for 2D-SWE + FIB-4). Based on the above findings, it is necessary to combine ATT with SWM, APRI, and FIB-4 to improve the diagnostic efficacy, especially in the assessment of early hepatocellular steatosis in patients with WD.

In this study, no correlation was found between ATT and TG, suggesting that TG cannot substitute ATT for early liver steatosis among patients with WD in clinical practice. In the guidelines for the diagnosis and treatment of NAFLD, WD was recognized as a secondary cause of liver steatosis [21]. Thus, monitoring ATT changes for patients with liver steatosis at follow-up is helpful in understanding the progression of the disease [22]. Currently, few studies have focused on the relationship between ATT and TG. In our study, TG did not differ in different groups of patients with WD. However, Chalasani N has pointed out that there could be a significant association between TG level and degree of liver steatosis in liver tissues [23], which was not consistent with the results, and the underlying reason may be attributed to the existence of patients with NAFLD in the study conducted by Chalasani N. Therefore, it is necessary to analyze clinical studies with a large sample size.

In addition, we found that there was a weak negative correlation between ATT and SWM, indicating that the level of SWM did not increase when ATT was at a high level, which was consistent with the fact that liver steatosis in patients with WD occurred before liver fibrosis, and it was no longer the main pathological change in the stages of liver fibrosis and cirrhosis, making SWM a noninvasive index for the assessment of liver fibrosis in patients with WD [24]. However, it remains to be clarified whether SWM can be used as a sensitive index for the assessment of early hepatic steatosis.

There were some limitations in this study. First, the clinical stratification of liver involvement was based on the levels of serological indexes for liver fibrosis rather than the results of liver biopsy due to its infeasibility in patients with WD with neurological impairments. Second, the WD patients included in this study were 5–18 years of age and experiencing rapid growth and development. Due to the limited sample size, the subgroup analysis based on their age was not carried out in the present study. However, we will continue to investigate this in the future.

## Conclusion

In summary, the ATT technique can be used for the non-invasive evaluation of early liver steatosis in children and adolescents with WD and has a high diagnostic efficacy. Furthermore, the combination of ATT with APRI, FIB-4, and SWM might have the potential to improve the clinical efficacy of the assessment compared to ATT alone.

## Data Availability

The datasets supporting the conclusions of this article are available from the corresponding author on reasonable request.

## Abbreviations

ATT: Attenuation coefficient
WD: Wilson’s disease
SWM: Shear Wave Measurement
APRI: AST to platelet ratio index
FIB-4: Fibrosis 4
CIV: Collagen type IV
HA: Hyaluronic acid
LN: Laminin
PIIINP: Precollagen type III N-terminal peptide
TG: Triglyceride
CP: Ceruloplasmin
ROC: Receiver operating characteristic
AUC: Area under the curve
2D: Two-dimensional
TE: Transient elastography
2D-SWE: Two-dimensional shear wave elastography
NAFLD: Nonalcoholic fatty liver disease
EASL: European Association for the Study of the Liver
PLT: Platelet counts
ALT: Alanine transaminase
AST: Aspartate transaminase
